# The Effects of Nuclear Factor Erythroid 2 (NFE2)-Related Factor 2 (Nrf2) Activation in Preclinical Models of Peripheral Neuropathic Pain

**DOI:** 10.3390/antiox11020430

**Published:** 2022-02-21

**Authors:** Paramita Basu, Dayna L. Averitt, Camelia Maier, Arpita Basu

**Affiliations:** 1 Pittsburgh Center for Pain Research and The Pittsburgh Project to End Opioid Misuse, Department of Anesthesiology & Perioperative Medicine, University of Pittsburgh School of Medicine, Pittsburgh, PA 15213, USA; 2 Division of Biology, School of the Sciences, Texas Woman’s University, Denton, TX 76204, USA; daveritt@twu.edu (D.L.A.); cmaier@twu.edu (C.M.); 3 Department of Kinesiology and Nutrition Sciences, School of Integrated Health Sciences, University of Nevada, Las Vegas, NV 89154, USA; arpita.basu@unlv.edu

**Keywords:** chemotherapy-induced peripheral neuropathy, chronic constriction injury, diabetic neuropathy, Nrf2, partial sciatic nerve ligation, peripheral neuropathy, rodents, sciatic nerve crush, spared nerve injury, spinal nerve ligation

## Abstract

Oxidative stress, resulting from an imbalance between the formation of damaging free radicals and availability of protective antioxidants, can contribute to peripheral neuropathic pain conditions. Reactive oxygen and nitrogen species, as well as products of the mitochondrial metabolism such as superoxide anions, hydrogen peroxide, and hydroxyl radicals, are common free radicals. Nuclear factor erythroid 2 (NFE2)-related factor 2 (Nrf2) is a transcription factor encoded by the *NFE2L2* gene and is a member of the cap ‘n’ collar subfamily of basic region leucine zipper transcription factors. Under normal physiological conditions, Nrf2 remains bound to Kelch-like ECH-associated protein 1 in the cytoplasm that ultimately leads to proteasomal degradation. During peripheral neuropathy, Nrf2 can translocate to the nucleus, where it heterodimerizes with muscle aponeurosis fibromatosis proteins and binds to antioxidant response elements (AREs). It is becoming increasingly clear that the Nrf2 interaction with ARE leads to the transcription of several antioxidative enzymes that can ameliorate neuropathy and neuropathic pain in rodent models. Current evidence indicates that the antinociceptive effects of Nrf2 occur via reducing oxidative stress, neuroinflammation, and mitochondrial dysfunction. Here, we will summarize the preclinical evidence supporting the role of Nrf2 signaling pathways and Nrf2 inducers in alleviating peripheral neuropathic pain.

## 1. Peripheral Neuropathic Pain and Erythroid 2 (NFE2)-Related Factor 2 (Nrf2)

In 1994, the International Association for the Study of Pain defined neuropathic pain as “pain initiated or caused by a primary lesion or dysfunction in the nervous system”. This definition was widely criticized due to the ambiguity of the term “dysfunction”. Some non-neurological diseases, such as fibromyalgia or irritable bowel syndrome that involve the central dysfunctions of the nociceptive system, could also fall under the definition of neuropathic pain. Therefore, a new definition was proposed in 2008 defining neuropathic pain as “initiated or caused by a primary lesion or dysfunction in the nervous system” [1,2]. The prevalence of neuropathic pain in the global population is estimated to be 6.9–10.0% [3], while in the United States, prevalence is estimated at 9.8% or 12.4% depending on whether the pain is diagnosed via clinical examination or self-reporting, respectively. However, it is difficult to accurately estimate neuropathic pain prevalence largely due to challenges in defining neuropathic pain and the variety of epidemiological assessment methods employed [4].

Damage or injury to the peripheral nerves is referred to as peripheral neuropathic pain [5]. Peripheral neuropathy is characterized by sensory ataxia, tingling, numbness, as well as muscle atrophy and weakness. When accompanied by neuropathic pain, sensations of “pins and needles”, burning, shooting, and/or stabbing pain are also experienced. Peripheral neuropathic pain can be either acquired, hereditary, or idiopathic and can result in a chronic neuropathic pain state. Common causes of neuropathic pain include postherpetic neuralgia, trigeminal neuralgia, diabetic neuropathy, chemotherapy-induced neuropathy, postsurgical neuropathic pain, spinal cord injury, and cancer. Diabetes is the major cause of acquired neuropathy; others include alcohol misuse, vitamin deficiencies, and immune-related factors [6]. Chronic neuropathic pain conditions incur significant financial burden for patients, the healthcare system, as well as society [4].

One mechanism underlying the development and maintenance of peripheral neuropathic pain is oxidative stress, which is an imbalance between the production of damaging free radicals and protective antioxidants, and unchecked free radicals begin damaging cells membranes, proteins, and DNA [7]. The most common free radicals belong to reactive oxygen species (ROS), reactive nitrogen species (RNS), and the products of the mitochondrial metabolism, such as superoxide anions, hydrogen peroxide, and hydroxyl radicals [8]. Mitochondrial dysfunction due to increased ROS is implicated in the pathogenesis of several neuropathies. In animal models of chemotherapy-induced neuropathy, paclitaxel induced swollen and vacuolated mitochondria in the axons of sensory nerves [9,10,11]. Under diabetic neuropathy, hyperglycemia reduced the threshold value for proton gradient in the mitochondrial membrane and increased superoxide production by continuous supply of proton donors from the tricarboxylic acid cycle [12,13]. Of note, oxidants can also be generated by enzymes in immune and non-immune cells [14] to provide protection against infections and environmental insults.

Once neuropathic pain has developed, it is a significant challenge to therapeutically manage. First-line treatments are often anticonvulsants and antidepressants; however, these are not effective for all neuropathic pain patients and come with unwanted negative side effects that make their use suboptimal. Thus, preclinical research is engaged in the discovery of novel neuropathic pain targets to inform the development of next-generation therapeutics to treat peripheral neuropathic pain with better efficacy and lesser side effects [15]. Targeting oxidative stress is one option being tested, as free radicals are known to cause tissue damage that may contribute to pain, such as disrupting the cellular signaling pathways that control cell division and migration, altering the production of proinflammatory and pronociceptive (pro-pain) mediators, and affecting numerous neural functions [15,16,17]. In support, crosstalk between ROS and RNS is a common feature underlying painful inflammatory diseases by leading to the activation of nuclear factor kappa B (NF-κB) and release of cytokines, lipid mediators, adhesion molecules, inflammasome assembly, and cyclooxygenase (COX)-2 [18,19,20].

Pharmacological and genetic studies report crosstalk between NF-κB and the transcription factor nuclear factor erythroid 2 (NFE2)-related factor 2 (Nrf2), such that the absence of Nrf2 can exacerbate NF-κB activity leading to the increased release of cytokine production. In turn, NF-κB can modulate the transcription and activity of Nrf2 [19]. Nrf2 is the product of the *NFE2L2* gene and a member of the cap ‘n’ collar subfamily of basic region leucine zipper (bZip) transcription factors. Nrf2 contains a bZip domain at the C-terminus that is responsible for the formation of heterodimers with other bZip proteins, such as small muscle aponeurosis fibromatosis (MAF) K, G, and F [21,22]. These heterodimers are the regulators of 250 human genes located at the regulatory enhancer sequence known as the antioxidant response element (ARE), which resembles the NFE2-binding motif [23].

During *normal physiological conditions*, Nrf2 is bound to Kelch-like ECH-associated protein 1 (Keap1) in the cytoplasm. Keap1 was identified as a Nrf2-binding protein by employing the yeast two hybrid system in which the inhibitory Neh2 domain of Nrf2 was a bait [24]. Keap1 contains two protein binding domains, the BTB (bric-a-brac, tramtrack, broad-complex) domain in the *N*-terminal region and Kelch repeats in the C-terminal region, which is homologous to *Drosophila* actin-binding protein Kelch (Kelch repeat, double glycine repeat domain) that mediates the binding of Keap1 to the Neh2 domain of Nrf2 [25,26,27]. The BTB domain is responsible for the homodimerization and binding of Keap1 to Cullin (Cul) 3, which is a scaffold protein of Nrf2 ubiquitin ligase (E3). Nrf2 has a half-life of approximately 20 min before it is degraded by proteasomes, which is mediated by the polyubiquitination through the Keap1/Cul3 ubiquitin ligase. Therefore, the protein levels of Nrf2 remain low in many cell types under normal physiological conditions [28,29,30].

**Figure 1:**
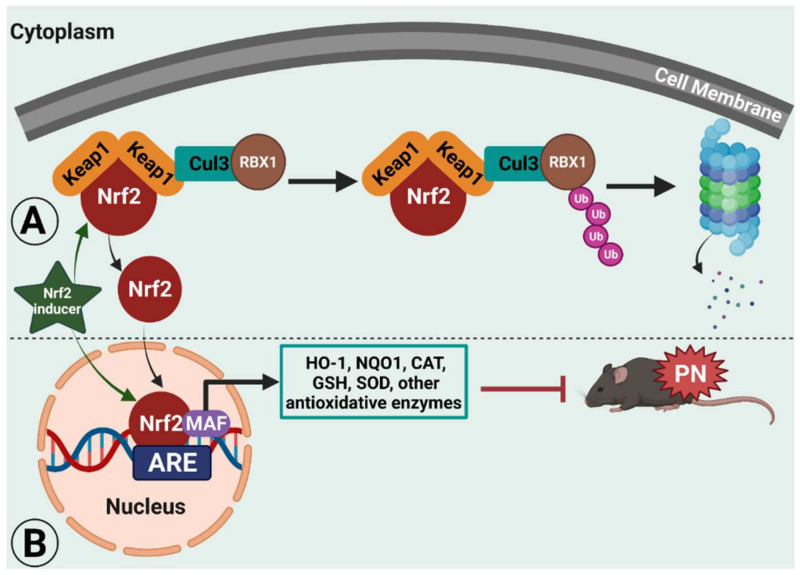
Illustrated working model of nuclear factor erythroid 2 (NFE2)-related factor 2 (Nrf2) signaling in rodent peripheral neuropathy (PN). Under normal physiological conditions, Nrf2 remains bound to Kelch-like ECH-associated protein 1 (Keap1) in the cytoplasm, which ultimately leads to proteasomal degradation. (**A**) Under PN, Nrf2 translocates to nucleus, where it heterodimerizes with muscle aponeurosis fibromatosis (MAF) and binds to antioxidant response element (ARE). This interaction with ARE leads to the transcription of several antioxidative enzymes that can ameliorate PN conditions in rodent models by inhibiting oxidative stress, neuroinflammation, and mitochondrial dysfunction. (**B**) Nrf2 inducers enhance the translocation of Nrf2 or its signaling to facilitate the transcription of antioxidative enzymes, ultimately leading to alleviation of neuropathic pain conditions. This figure was created using BioRender.

During *stressful physiological conditions*, Nrf2 is released from Keap1 and translocates into the nucleus where it heterodimerizes with the MAF proteins. The complex Nrf2–MAF binds to ARE, initiating the transcriptions of several cytoprotective genes, such as heme oxygenase-1 (HO-1), NAD(P)H:quinone oxidoreductase1 (NQO1), superoxide dismutase (SOD), glutathione cysteine ligase, glutathione S-transferases, and catalase (Figure 1) [31,32,33]. Thus, Nrf2 provides protection against oxidative stress. In support, Nrf2 knockout mice are highly susceptible to oxidative stress-related chemical toxicity and disease [34,35,36,37,38,39,40,41], leading researchers to postulate that targeting Nrf2′s protective role against oxidative stress and mitochondrial dysfunction [42,43,44,45] may provide a novel target for alleviating neuropathic pain. Here, we discuss pre-clinical evidence across several animal models of neuropathic pain of the therapeutic potential of targeting Nrf2 signaling and Nrf2 inducers (chemical structures are illustrated in Table 1).

## 2. Nrf2 Signaling and Its Inducers in Different Peripheral Neuropathic Pain Conditions

### 2.1. Diabetic Neuropathy (DN)

Diabetes is one of the most debilitating conditions in patients, affecting a large world population. The adult population affected by diabetes is projected to increase by 170% by 2030 [46]. DN is one of the most common complications of diabetes and is defined as “a demonstrable disorder, either clinically evident or subclinical, that occurs in the setting of diabetes mellitus without other causes for peripheral neuropathy” [47]. The pathophysiological mechanisms that cause hyperglycemia-induced diabetic neuropathy include advance cellular glycation end products, such as carboxy-methyl lysine, glyoxal-derived lysine, carboxy-ethyl lysine, methyl-glyoxal-derived lysine dimer, pyralline and deoxy-glycosome-derived lysine, hexosamine pathway, oxidative–nitrosative stress, neuroinflammation, protein kinase C activation, and sorbitol accumulation [48,49,50].

Under a reducing environment, the NF-κB remains inactive. However, the presence of oxidative/nitrosative stress leads to the activation of NF-κB by promoting phosphorylation and degradation of IκB [51]. On the other hand, Nrf2 increases the levels of intracellular glutathione (GSH) and GSH-dependent enzymes and, thus, favors the reducing environment, which promotes the inhibition of NF-κB [52]. The expression of Nrf2 and HO-1 was downregulated in the sciatic nerves of diabetic animals compared to the control animals [53]. Furthermore, Nrf2-deficient mice exhibited greater induction of NF-κB-induced proinflammatory genes, such as interleukins, tumor necrosis factor (TNF-α), inducible nitric oxide synthase, and COX-2 [54]. Liu et al. reported that NF-κB activation led to the transcriptional repression of Nrf2 [55]. They reported that the NF-κB p65 subunit promoted the localization of transcription repressors, histone deacetylases with Nrf2/ARE, and sequestered coactivators such as CREB binding protein and, therefore, repressed the beneficial effects of *NRF2* [55]. Yu et al. also reported that the N-terminal region of the NF-κB p65 subunit bound to Keap1 and exerted an additional mechanism of Nrf2–ARE inhibition [56]. On the other hand, HO-1 inhibited the TNF-α dependent activation of NF-κB in the endothelial cells [57]. Taken together, these data suggest a crosstalk between NF-κB and Nrf2 transcription factors, affecting signaling pathways that maintain the cellular redox homeostasis.

The vascular complications of diabetic neuropathy involve impaired endoneurial blood flow to the peripheral nerves, leading to the destruction of neurons and Schwann cells (SCs) [58]. Overexpression of Nrf2 may repair nerve injury as Tang et al. reported that SCs overexpressing Nrf2 restored nerve conduction velocity, myelin sheath thickness, and sciatic vasa nervorum while also inhibiting hyperglycemia-induced apoptosis [59]. Further, overexpressing Nrf2 promoted angiogenesis by regulating the Toll-like receptor 4/NF-κB signaling pathway in a rodent model of diabetic neuropathy [59].

Table 2 summarizes the effects of different Nrf2 inducers in rodent models of diabetic neuropathy. Tangluoning, a traditional Chinese medicine, downregulated the phosphorylated protein kinase RNA-like endoplasmic reticulum kinase (p-PERK) in SCs and upregulated the Nrf2 expression resulting in decreased ROS and apoptosis due to ROS [60]. In addition, paeoniflorin, the main active ingredient of peony, also decreased SC apoptosis via the PERK/Nrf2 pathway [61]. A large variety of natural products can suppress the expression of NF-κB and other proinflammatory and apoptotic markers, including bergenin [62], diosgenin [63], deguelin [64], fisetin [65], anomalin [66], resveratrol [67], rutin [68], taurine [69], diphenyl diselenide [70], tanshinone IIA (one of the most active components of the traditional Chinese herbal medicine *Danshen*) [71], as well as Nrf2 inducers bardoxolone methyl [72] and oltipraz [73]. These natural products can also enhance the expression of Nrf2, as well as other antioxidative enzymes (HO-1, GLUT1,3), by activating Nrf2 signaling pathways in diabetic animals.

Another Nrf2 inducer, polydatin (a stilbenoid glucoside and major resveratrol derivative), enhanced the silent information regulator-1 (sirtuin, SIRT1) and Nrf2 axis in rats with diabetic neuropathy [74]. Activation of SIRT1, which is a nicotinamide adenosine dinucleotide (NAD+)-dependent deacetylase, recuperates nerve function by activating mitochondrial biogenesis [75]. SIRT1 deacetylates Nrf2 that leads to the transcription of antioxidative enzymes (NQO-1, HO-1, SOD) [76,77]. Polydatin increased the activation of SIRT1 that resulted in deacetylation of peroxisome proliferator-activated receptor-gamma coactivator-1α (PGC-1α), which facilitated mitochondrial biogenesis and Nrf2 signaling [74]. In addition, isoliquiritigenin significantly activated SIRT1 with the concurrent increase in PGC-1αmediated mitochondrial biogenesis and enhanced Nrf2-directed antioxidant signaling [78].

Combination treatment with rutin and the COX-2 inhibitor nimesulide synergistically attenuated oxidative damage, reduced inflammatory mediators, and reduced mitochondrial ROS production while restoring Nrf2/HO-1 in the sciatic nerve of a rat model of diabetic neuropathy [79]. Quercetin, in combination with cinnamaldehyde or hirudin, downregulated the expression of the proinflammatory markers NF-κB, interleukin-6 (IL-6), and TNF-α while upregulating the expression of Nrf2/HO-1 [80]. Administration of another Nrf2 inducer sulforaphane, alone and/or in combination with delta opioid receptor (DOR) agonists (dPen(2),d-Pen(5))-Enkephalin) (DPDPE) and (+)-4-((α(R)-α-((2S,5R)-4-allyl-2,5-dimethyl-1-piperazinyl)-(3-methoxybenzyl)-N,N-diethylbenzamide) (SNC-80), enhanced the antinociceptive activity of DOR in parallel with enhancement of HO-1 protein and inhibition of JNK phosphorylation in diabetic mice [81]. These studies demonstrate that Nrf2 inducers could serve as adjunctive therapies with currently used treatments of peripheral neuropathic pain.

**Table 2:** Evidence of the effects of Nrf2 inducers in rodent models of diabetic neuropathy (DN).

Nrf2 Inducer	Animals (Sex, Strain)	Dose (mg/kg) and Administration Route	Mechanism of Action	Reference
Resveratrol	Nrf2^−/−^ and Nrf2^+/+^ CD1/ICR mice	10 mL/kg 10%, intragastric	Nrf2 pathway	[67]
Polydatin	Male Sprague Dawley rats	25 and 50 mg/kg, oral 10, 20 µM in neuro2a—mouse neuroblastoma cells—in vitro	SIRT1/Nrf2 pathway	[74]
Oltipraz	Rat Schwann cell line	20 µM on RSC96 cells—Schwann cell line—in vitro	Nrf2/NQO1 pathway	[73]
Bardoxolone methyl	Male Sprague Dawley rats	15 and 30 mg/kg/day, oral	keap1-Nrf2-ARE pathway	[72]
Diosgenin	Male C57 mice	50 and 100 mg/kg, intragastric	Nrf2/HO-1 pathway	[63]
Bergenin	Male C57BL/6 mice	3.125–25 mg/kg, i.p.	Nrf2 pathway	[62]
Diphenyl diselenide	Male Sprague Dawley rats	5 and 15 mg/kg, oral	Nrf2/Keap1 pathway	[70]
Deguelin	Male Sprague Dawley rats	4, 6, and 8 mg/kg, oral gavage	Nrf2 pathway	[64]
Tanshinone IIA	Male Sprague Dawley rats	25 mg/kg, i.p.	Nrf2/ARE pathway	[71]
Rutin	Male Wistar rats	Rutin—100 and 200 mg/kg, i.p. Nimesulid—5 and 10 mg/kg, i.p. Combination—200 mg/kg rutin + 10 mg/kg nimesulid, i.p.	Nrf2/HO-1/NF-κB and COX pathway	[79]
Sulforaphane	BKS.Cg-m+/+Leprdb/J and db/db mice	Sulforaphane—2.5, 5, and 10 mg/kg, s.c. DPDPE and SNC-80—0.15, 0.5, 1, and 5 mg/ kg, s.c. Combination—10 mg/kg, i.p. sulforaphane alone or 10 mg/kg, i.p. sulforaphane + 0.15 mg/kg, s.c DPDPE, 10 mg/kg, i.p. sulforaphane + 0.5 mg/kg, s.c. SNC-80	Nrf-2/HO-1 pathway	[81]
Quercetin	Sprague Dawley rats’ embryos	10 mmol/L quercetin, 1 IU/mL hirudin, 100 nmol/L cinnamaldehyde on DRG neurons from 15d embryos of Sprague Dawley rats—in vitro Combination—quercetin + hirudin, quercetin + cinnamaldehyde, cinnamaldehyde + hirudin, quercetin + cinnamaldehyde + hirudin	Nrf-2/HO-1 pathway	[80]
Tangluoning	Male Sprague Dawley rats	10.9 g and 21.8 g crude drug/kg/day, intragastric	PERK/Nrf2 pathway	[55]
Isoliquiritigenin	Male Sprague Dawley rats	10 and 20 mg/kg, oral 2.5 and 5 µM on neuro2a—mouse neuroblastoma cells—in vitro	SIRT1/Nrf2 pathway	[78]
Anomalin	Male ICR mice	50 mg/kg, i.p. 50 µM on DRG primary cells, N2a—mouse neuroblastoma cell line	Nrf2 pathway	[66]
Fisetin	Male Sprague Dawley rats	5 and 10 mg/kg, oral	Nrf2 pathway	[65]
Rutin	Male Sprague Dawley rats	5, 25, and 50 mg/kg, i.p.	Nrf2 pathway	[68]
Paeoniflorin	Rat Schwann cell line	1, 10, and 100 µM in RSC96 cells—Schwann cell line—in vitro	Nrf2/ARE pathway	[61]
Taurine	Male Wistar rats	2% *w*/*v*, oral	Nrf2/HO-1 pathway	[69]

Note. Table is organized based on the most recent to oldest publications.

### 2.2. Chemotherapy-Induced Peripheral Neuropathy (CIPN)

Chemotherapy-induced peripheral neuropathy (CIPN) is one of the most common neuropathies caused by antineoplastic agents [82], including platinum-based chemotherapeutic agents, taxanes, thalidomide and its analogues, and ixabepilone [83]. Oxaliplatin and paclitaxel are known to induce severe neuropathy during or immediately after the drug infusion [84]. The common symptoms of CIPN include “pins-and-needles” sensations, heat, burning, pain, as well as motor discoordination and muscle weakness [85]. Oxaliplatin is a third-generation platinum-derived chemotherapeutic agent that is used to treat colorectal and other cancers [86]. However, the beneficial effects of oxaliplatin and paclitaxel must be weighed against the risk of neurological side effects and peripheral neuropathic disorders, which affect 85–95% of the patients exposed to paclitaxel [87,88].

The effects of other Nrf2 inducers in rodent models of CIPN have been summarized in Table 3. *Nrf2^−/−^* knockout mice display severe mechanical and cold hypersensitivities. Yang et al. reported that oxaliplatin-induced neuropathy in *Nrf2^−/−^* knockout mice resulted in greater production of ROS, decreased mitochondrial membrane potential with abnormal release of intracellular calcium, higher cytochrome C-related apoptosis, and overexpression of transient receptor potential (TRP) ion channels. All of these effects were attenuated by activating Nrf2 signaling with the Nrf2 inducer sulforaphane [89]. Similarly, Miao et al. reported that paclitaxel impaired Nrf-ARE and SOD in the dorsal root ganglia (DRG) in parallel with the production of oxidative stress markers (8-isoprostaglandin F2α (8-iso PGF2α) and 8-hydroxy-2′-deoxyguanosine (8-OHdG)) and proinflammatory cytokines (interleukin-1 beta (IL-1β), IL-6 and TNF-α), likely contributing to neuropathic pain [90]. Again, activation of the Nrf2/HO-1 signaling pathway alleviated paclitaxel-induced neuropathic pain, with a single dose of oltipraz attenuating pain while repeated administration abolished pain [91]. Remarkably, the antinociceptive effect of oltipraz was reversed by the Nrf2 inhibitor trigonelline, implicating a major role for Nrf2 signaling in chemotherapy-induced neuropathic pain.

Interactions with other inflammatory mediators have also been implicated. Crosstalk between the peroxisome proliferator-activated receptor gamma (PPARγ) and Nrf2/HO-1 signaling pathway has also been reported and may inform therapy development. Zhou et al. found the PPARγ selective agonist rosiglitazone reduced pain and upregulated the expression of Nrf2 and HO-1 in the spinal cord of paclitaxel-treated rats [92]. Moreover, the analgesic activity of rosiglitazone was abolished by the application of the Nrf2 inhibitor trigonelline. In addition, the Nrf2-ARE signaling pathway can also be targeted by microRNA (miRNA) treatment. Treatment with an inhibitor of miR-155, a miRNA that regulates inflammation, restored the oxaliplatin-induced impairment of the Nrf2-antioxidant response and Nrf2-regulated NQO1 protein expression in the dorsal horn of male rats [93]. Inhibition of miR-155 led to the attenuation of NOX4 protein expression, other oxidative stress products (8-iso PGF2α/8-OHdG), and TRPA1 in the dorsal horn of oxaliplatin-treated rats [93]. These data support the potential inhibitory effect of miR-155 in chemotherapy-induced PN via the Nrf2-ARE signaling pathway.

Alkaloid levo-corydalmine [94], antioxidant alphalipoic acid [95], mitoquinone [96], endogenous fatty acid amide palmitoylethanolamide (PEA) in association with oxazoline forming the 2-pentadecyl-2-oxazoline of palmitoylethanolamide (PEA-OXA) [97], curcumin [98], quercetin [99], resveratrol [100], formononetin [101], berberine [102], dimethyl fumarate and its metabolite monomethyl fumarate [103], and oleuropein [104] can reduce pain and inflammation associated with CIPN by activating the Nrf2 pathway. In addition to these Nrf2 inducers, Zhang and Xu shed light on the effects of bromodomain-containing protein 4 (BRD4) on the alleviation of vincristine-induced PN [105]. The study reported that BRD4 inhibition significantly reduced oxidative stress in sciatic nerve tissues by activating Nrf2. They postulate that the reducing expression of BRD4 by genetic therapy or drug intervention could inhibit macrophage infiltration and reduce inflammation and oxidative stress, providing a novel therapeutic target to be developed for the treatment of CIPN.

In addition to the aforementioned preclinical studies, Zhao et al. reported that electroacupuncture intervention restored the impairment of Nrf2-ARE/SOD, Nrf2-regulated NQO1, inhibited oxidative stress products (8-iso PGF2α/8-OHdG), and thereby attenuated the mechanical and thermal hypersensitivities in rats treated with paclitaxel [106]. Therefore, the study confirms the use of electropuncture as an alternative treatment strategy to treat CIPN [106].

**Table 3:** Evidence of the effects of Nrf2 inducers in rodent models of chemotherapy-induced peripheral neuropathy (CIPN).

Nrf2 Inducer	Animals (Sex, Strain)	Dose (mg/kg), Route of Administration	Mechanism of Action	Reference
PEA-OXA	Male Wistar rats	10 mg/kg, oral	NF-κB/Nrf-2 pathway	[97]
Oleuropein	Male Wistar rats	Oleuropein—20 mg/kg, oral Combination—Oleuropein—20 mg/kg, oral + suvorexant—an orexin receptor antagonist—20 mg/kg, oral	Nrf2 pathway	[104]
Curcumin	Male Sprague Dawley rats	100 and 200 mg/kg, oral	Nrf2/HO-1 pathway	[98]
Mitoquinone	Male ICR mice	2.5, 5 and 10 mg/kg, intragastric	Nrf2 pathway	[96]
Formononetin	Male C57BL/6 mice	10 mg/kg, i.p. 10 µM on mouse ND7/23 neuron cells, colon cancer cells (CT-26), human colorectal carcinoma cells (Caco-2, DLD-1, and HCT-116), human lung adenocarcinoma cells (PC9, A649, H1975, and HCC8827), human lung squamous cell carcinoma cells (H520), and human pancreatic cancer cells (BxPC3 and Panc1)—in vitro	Keap1-Nrf2-GSTP1 pathway	[101]
Resveratrol	Male Sprague Dawley rats	7 and 14 mg/kg, oral	Nrf2/HO-1 pathway	[100]
Quercetin	Male Sprague Dawley rats	25 and 50 mg/kg, oral	Nrf2/HO-1 pathway	[99]
Oltipraz	Male Sprague Dawley rats	10, 50, 100 mg/kg/day, i.p.	Nrf2/HO-1 pathway	[91]
Rosiglitazone	Male Sprague Dawley rats	5, 25, and 50 mg/kg, i.p.	Nrf2/HO-1 pathway	[92]
Levo-corydalmine	Male ICR mice	5, 10, and 20 mg/kg, intragastric	Nrf2/HO-1/CO pathway	[94]
Berberine	Male Wistar rats	10 and 20 mg/kg, i.p.	Nrf2 pathway	[102]
Alphalipoic acid	Male Sprague Dawley rats	15, 30, and 60 mg/kg, i.p.	Nrf2 pathway	[95]
L-carnosine	Male and female Egyptian patients	500 mg, oral in patients—clinical trial	Nrf2 pathway	[107]
Dimethyl fumarate and its metabolite monomethyl fumarate	Rat	0.3, 1, 3, or 10 mM dimethyl fumarate or monomethyl fumarate on PC12 cell—a rat pheochromocytoma cell	Nrf2 pathway	[103]
Sulforaphane	Nrf2^+/+^ and Nrf2^−/−^ C57BL/6 mice	5 mg/kg, i.p. 10 µM on DRG neurons	Nrf2 pathway	[89]

Note. Table is organized based on the most recent to oldest publications.

### 2.3. Other Peripheral Nerve Injury Models

#### 2.3.1. Sciatic Nerve Chronic Constriction Injury (CCI)

Chronic constriction injury (CCI) of the sciatic nerve is one of the most widely used chronic neuropathic in vivo pain models, resembling the pain found in humans [108]. The effects of Nrf2 inducers in rodent models of CCI are summarized in Table 4. Total and nuclear Nrf2 and its downstream protein HO-1 are increased in spinal microglia following CCI [109]. Hydrogen sulfide (H_2_S) is known to be involved in the pathogenic processes of inflammation, heart failure, neurodegenerative diseases, and sepsis [110]. H_2_S also confers protective effects against neuropathic pain by inhibiting inflammatory responses and microglial activation [111]. Chen et al. reported that NaHS (a common donor for H_2_S) increased Nrf2 and HO-1 and inhibited NF-κB and ionized calcium-binding adaptor molecule 1 (Iba1; microglia marker) in the spinal cord of CCI-induced rats. However, inhibition of the Nrf2 expression by siRNA aggravated CCI-induced allodynia and hyperalgesia and NaHS did alleviate CCI-induced hypersensitivities following siRNA treatment. Blockage of Nrf2 (by siRNA) and of HO-1 (by Sn-protoporphyrin-IX) partially reduced NaHS-induced inhibition of cytokines TNFα, IL-1β, IL-6 and high mobility group box 1 in CCI-induced rats [109]. Together, these results indicate that NaHS exerts anti-inflammatory activity via activating the Nrf2/HO-1 signaling pathway.

Wang et al. [112] reported that two organosulphur compounds of garlic oil, such as diallyl disulfide and diallyl trisulfide, restored the brain-derived neurotrophin factor (BDNF), H_2_S, and Nrf2 in the sciatic nerve and DRG of CCI-subjected rats. Administration of a BDNF blocker abolished the diallyl disulfide and diallyl trisulfide-induced reduction in pain sensitivity and reduced the levels of BDNF and Nrf2. However, the BDNF blocker ANA-12 did not affect H_2_S levels, indicating that BDNF is the downstream mediator of H_2_S. In conclusion, Wang et al. provide strong evidence of the involvement of H_2_S-BDNF-Nrf2 signaling in CCI-induced neuropathic pain [112].

In addition to the active constituents of garlic oil, several other natural compounds, including bromelain [113], a major protease from pineapple (*Ananas comosus*) [114], oleuropein, [104], paeoniflorin [115], a bioactive compound isolated from the dried roots of Paeonia *lactifora* Pallas or *P. veitchii* Lynch [115], the extracts from *Thymus algeriensis* and *T. fontanesii* (*Lamiaceae*) [116], quercetin [117], plumbagin [118], poly (ADP-ribose) polymerase (PARP) inhibitor 4-amino 1, 8-naphthalimide (4-ANI) [117], DOR agonist UFP-512 [119], and RTA-408 (a novel synthetic triterpenoid under clinical investigation) [120] ameliorated CCI-induced neuropathic pain. Targeted mechanisms reported involved either increasing the Nrf2 activity [104,114,116,117,119,120], enhancing the translocation of Nrf2 while suppressing the spinal NOD-like receptor protein 3 (NLRP3) inflammasome and NF-κB [115], or inhibiting PARP activation [117]. For example, Arruri et al. reported that carvacrol attenuated CCI-induced neuropathic pain by inhibiting NLRP3 and activating autophagy via the Keap1/Nrf-2/p62 forward-feedback loop and augmenting mitochondrial quality control (MCQ) [121,122]. To ensure cell homeostasis, MCQ operates through the coordination of various processes, including proteostasis, biogenesis, dynamics, and mitophagy operates [123].

Oltipraz, a previously noted Nrf2 inducer, alleviated neuropathic pain by inducing the Nrf2/HO-1/NQO1 signaling pathway in the spinal cord of CCI mice. Further, oltipraz-induced antidepressant activity in CCI-induced animals could be attributed to the induction of the Nrf2/HO-1/NQO1 signaling pathway in the hippocampus and/or prefrontal cortex of the CCI-subjected animals [124]. The study also confirmed that oltipraz potentiated the overexpression of NQO1 in the prefrontal cortex, which is similar to the activity of other antidepressants such as desipramine [125]. Therefore, these data confirm the involvement of NQO1 in the antidepressant effects of oltipraz [124]. Dexmedetomidine, which is a highly selective sedative, anxiolytic, and analgesic α2-adrenergic agonist [126,127], also alleviated CCI-induced neuropathic pain. Dexmedetomidine was found to downregulate Keap1, upregulate Nrf2 and HO-1, inhibit inflammation and apoptosis [128], and suppress NLRP3 through activating Nrf2 [129]. In contrast, Riego et al. (2018) reported that a carbon monoxide-releasing compound (tricarbonyldichlororuthenium(II) dimer) or the HO-1 inducer cobalt protoporphyrin IX (CoPP) did not alter Nrf2 and NQO1, but did increase the expression of HO-1 [130]. Shan et al. [131] reported that CoPP enhanced Nrf2 activity in the human hepatoma cell line Huh-7, indicating that the potency of CoPP varies from in vivo to in vitro application. These findings suggest that CoPP might be metabolized quickly, and therefore its efficacy is reduced in animals. To enhance its therapeutic efficiency, future studies should focus on delivering CoPP via different formulations, such as neon-particles, nanovesicles, emulsions, prodrugs, derivatives, and other forms, which will enhance its bioavailability in the in vivo system.

The efficacy of Nrf2 to potentiate the antinociceptive activity of opioids has also been reported [132,133]. Nrf2 inducers sulforaphane [132] and 5-fluoro-2-oxindole [133] alleviated CCI-induced pain hypersensitivities and diminished the anxiety- and depressive-like behaviors associated with persistent neuropathic pain in mice. Sulforaphane normalized oxidative stress by inducing Nrf2/HO-1 signaling, reducing the activation of microglia, and preventing CCI-induced phosphorylation of c-Jun N-terminal kinase (JNK) and extracellular signal-regulated kinase (ERK)1/2, p-38 in spinal cord and/or hippocampus and prefrontal cortex. Similar to sulforaphane, 5-fluoro-2-oxindol reduced microglia activation and increased activation of Nrf2/HO-1/NQO1 signaling in the spinal cord and/or hippocampus [133]. Both sulforaphane and 5-fluoro-2-oxindol potentiated the antinociceptive activity of morphine by normalizing the downregulation of mu-opioid receptor. Wang and Wang reported that sulforaphane attenuated CCI-induced pain hypersensitivities, reduced pro-inflammatory cytokines, counteracted CCI-induced enhancement of COX2 and iNOS and increased anti-inflammatory cytokines [134]. Moreover, sulforaphane treatment increased the expression of mu-opioid receptors in CCI mice, confirming the antinociceptive and anti-inflammatory activities of sulforaphane against CCI-induced neuropathy [134]. Together, these studies support the beneficial effects of Nrf2 inducers in the management of persistent neuropathic pain and the associated comorbidities along with the improvement of the analgesic properties of morphine in rodent models of neuropathic pain.

In addition to the natural inducers of Nrf2, physical activity has also been attributed to the translocation of Nrf2 in neuropathic pain. Green-Fulgham et al. reported that 6 weeks of voluntary wheel running increased the translocation of Nrf2 at the site of sciatic nerve injury, whereas 3 weeks of wheel running exerted no effects on the translocation of Nrf2 and reduction in neuropathic pain, which indicates the importance of long-term physical activity in alleviating neuropathic pain [135] and could also be an important adjunctive therapy when considering Nrf2 for neuropathic pain relief.

**Table 4:** Evidence of the effects of Nrf2 inducers in a rodent model of chronic constriction injury (CCI).

Nrf2 Inducer	Animals (Sex, Strain)	Dose (mg/kg), Route of Administration	Mechanism of Action	Reference
Carvacrol	Male Sprague Dawley rats	30 and 60 mg/kg, oral	Keap1/Nrf-2/p62 pathway	[122]
Oleuropein	Male Wistar rats	Oleuropein—10 and 20 mg/kg, oral Combination—Oleuropein—20 mg/kg, oral + suvorexant—an orexin receptor antagonist—10 mg/kg, oral Oleuropein—20 mg/kg, oral + suvorexant—20 mg/kg, oral	Nrf2 pathway	[104]
5-fluoro-2-oxindole	Male C57BL/6J mice	10 mg/kg, i.p.	Nrf2/HO-1/NQO1 pathway	[133]
Dexmedetomidine	Male Sprague Dawley rats	15 g/kg at 5 g/kg/h, i.p.	Keap1–Nrf2–HO-1 pathway	[128]
Dexmedetomidine	Male Sprague Dawley rats	1, 2, and 5 µg/kg, i.p.	NLRP3/Nrf2 pathway	[129]
RTA-408	Male C57BL/6J mice	1, 5, and 10 μg, i.t.	Nrf2 pathway	[120]
Bromelain	Male Wistar rats	30 and 50 mg/kg, oral	Nrf2 pathway	[114]
Paeoniflorin	Male Sprague Dawley rats	25, 50, and 100 mg/kg, i.p.	Keap1-Nrf2 pathway	[115]
*Thymus algeriensis* and *T. fontanesii* extracts	Male Wistar rats	200 and 400 mg/kg, oral	Nrf2 pathway	[116]
Diallyl disulfide and diallyl trisulfide	Male Wistar albino rats	Diallyl disulfide—25 and 50 mg/kg, oral Diallyl trisulfide—20 and 40 mg/kg, oral	H_2_S-BDNF-Nrf2 pathway	[112]
NaHS (a common donor for H_2_S)	Male Sprague Dawley rats	15, 30, 60 mg/kg, abdominal cavity administration	Nrf2/HO-1 pathway	[109]
Oltipraz	Male C57BL/6J mice	10 mg/kg, i.p.	Nrf2/HO-1/NQO1 pathway	[124]
UFP-512	Male C57BL/6J mice	UFP-512—1, 3, 10, 20, and 30 mg/kg i.p. Combination—1 mg/kg, i.p. UFP-512 + sulforaphane—10 mg/kg, i.p.	Nrf2/HO-1 pathway	[119]
Sulforaphane	Male C57BL/6J mice	10 mg/kg, i.p.	Nrf2/HO-1/NQO1 pathway	[132]
Plumbagin	Male Sprague Dawley rats	10 and 20 mg/kg, oral	Nrf2 pathway	[118]
Quercetin + PARP inhibitor-4-ANI	Male Sprague Dawley rats	Quercetin—25 mg/kg, oral 4-ANI—3 mg/kg, oral Combination—quercetin + 4-ANI	Nrf2 pathway	[117]
Sulforaphane	Male C57BL/6J mice	0.1–100 mg/kg, i.p.	Nrf2 pathway	[134]

Note. Table is organized based on the latest to oldest publication year of the articles.

#### 2.3.2. Sciatic Nerve Crush (SNC) Injury

Sciatic nerve crush (SNC) in rodents represents an axonotmesis-like moderate PN, characterized by the degradation of the myelin sheath and Wallerian degeneration of the distal ends of axons [136]. Zhang et al. reported impaired functional recovery in Nrf2^−/−^ mice compared to the wildtype (WT) mice in a SNC model. Furthermore, the study reported the presence of larger myelin debris with less accumulation of macrophages in Nrf2^−/−^ mice. The axonal regeneration in Nrf2 knockout mice was slower as compared to the WT. Even after 3 months of SNC injury, the Nrf2 knockout mice showed more thinly myelinated axon fibers compared to the WT mice. Together, these data support the therapeutic intervention with Nrf2 inducers for SNC injury [137]. In a similar study with Nrf2 knockout mice, promotion of reprogramming and proliferation of Schwann cells and inhibition of myelination and redifferentiation of repair Schwann cells was observed and treatment with the Nrf2 inducer dimethyl fumarate did not affect the myelination of regenerated nerves. This study provides insights into the molecular mechanism for Schwann cell plasticity, as well as the importance of the Nrf2-antioxidant system in SNC injury [138]. Qiu et al. reported that isoquercitrin upregulated Nrf2 and SOD while downregulating NAD+, Nox4, and dual oxidase 1, thus suppressing the oxidative stress following SNC [139]. Low doses of curcumin (0.2 mg/day for 4 weeks) [140] or sesame oil (0.5, 1 and 2 mL/kg for 6 days; oral administered) [141] showed antioxidant activity by significantly upregulating the expression of Nrf2 and decreasing ROS production and lipid peroxidation in a rodent model of SNC injury [140,141]. These results indicate that natural Nrf2 inducers can alleviate neuropathic pain sensitivity by decreasing different oxidative stress markers following SNC injury (Table 5).

**Table 5:** Evidence of the effects of Nrf2 inducers in rodent models of sciatic nerve crush (SNC), partial sciatic nerve ligation (PSNL), spared nerve injury (SNI), and spinal nerve ligation (SNL).

Nrf2 Inducer	Animals (Sex, Strain)	Dose (mg/kg), Route of Administration	Mechanism of Action	Reference
**Sciatic nerve crush (SNC) injury**				
Isoquercitrin	Male ICR mice	20 mg/kg/day, i.p. 40 to 320 μM isoquercitrin on primary Schwann cells isolated from sciatic nerves of neonatal 1-day-old Sprague Dawley rats—in vitro	Nrf2 pathway	[139]
Curcumin	Male Sprague Dawley rats	0.2 mg/day, continuous delivery through mini-osmotic pumps	Nrf2 pathway	[140]
Sesame oil	Male SPF C57BL/6 mice	0.5, 1 and 2 mL/kg, oral	Nrf2 pathway	[141]
**Partial sciatic nerve ligation (PSNL)**				
ECN	Male albino mice	1 and 5 mg/kg, i.p.	Nrf2/HO-1/NQO1 pathway	[142]
**Spared nerve injury (SNI)**				
tBHQ	Male Sprague Dawley rats	1 and 10 μM, i.t.	Nrf2 pathway	[143]
Dimethyl fumarate	Male Sprague Dawley rats and male and female wild type and Nfe2l2^−/−^ mice	30, 100, and 300 5 mL^−1^ kg^−1^, oral	Nrf2 pathway	[144]
Sulforaphane	Male Sprague Dawley rats	30 mg/kg, i.p.	Keap1-Nrf2 signaling	[145]
**Spinal nerve ligation (SNL)**				
Dimethylitaconate	Male C57BL/6 mice	10 mg or 20 mg, i.p. 250 µM on BV2 microglial cell line	Nrf2 pathway	[146]

Note. Table is organized based on the most recent to oldest publications.

#### 2.3.3. Partial Sciatic Nerve Ligation (PSNL)

In animal models of partial sciatic nerve ligation (PSNL), the dorsal third to half of the common sciatic nerve at the upper thigh level is ligated [147]. Although studies have reported the involvement of oxidative stress in PSNL-induced neuropathic pain [148,149,150,151], involvement of Nrf2 inducers or Nrf2 signaling pathways in a rodent model of PSNL has not been studied extensively. A study by Khan et al. demonstrated that sesquiterpenoid 7β-(3-Ethyl-*cis*-crotonoyloxy)-1α-(2-methylbutyryloxy)-3,14-dehydro-*z*-notonipetranone (ECN) from *Tussilago farfara* (Asteraceae) and commercially available pregabalin significantly enhanced Nrf2, HO-1, and NQO1 mRNA in the rodent sciatic nerve and spinal cord. In addition, ECN treatment also upregulated Nrf2 protein expression in mice subjected to PSNL (Table 5) [142]. Together, these data provide evidence for the potential pain-alleviating effect of ECN in PSNL-like injuries.

#### 2.3.4. Spared Nerve Injury (SNI)

The spared nerve injury (SNI) model involves the selective injury of peroneal and tibial nerves, leaving the sural nerve intact [152]. The model produces hypersensitivity at the location of the spared sural nerve that is comparable to the stimulus-evoked pain observed under clinical settings of neuropathic pain syndromes in humans [153]. Zhao et al. reported the involvement of Sirt2 in modulating the Nrf2 signaling pathway and oxidative stress in animals subjected to SNI. Sirtuins are a family of NAD^+^-dependent histone deacetylases that are also involved in protein acetylation and deacetylation besides histone deacetylation [154], as well as promotion of antioxidants and inhibition of ROS in mammalian cells [155]. In Zhao et al.’s study, SNI decreased Sirt2, Nrf2, and its target gene NQO1 in the spinal cord of rats. Intrathecal administration of the Nrf2 agonist tBHQ reversed the SNI-induced decrease in oxidative stress markers and alleviated SNI-induced mechanical and thermal hypersensitivities, leading to the normalization of Nrf2 and NQO1 expression in the spinal cord. Furthermore, administration of a recombinant adenovirus expressing Sirt2 increased Sirt2 levels and restored expressions of Nrf2 and NQO1, leading to the amelioration of SNI-induced pain sensitivity [143].

Nrf2 inducer dimethyl fumarate alleviated SNI-induced hyperalgesia, increased superoxide dismutase activity, reduced the SNI-induced increase in chemokines and cytokines (including IL-1β), and resolved the mitochondrial dysfunction and oxidative stress that drove the nociceptive hypersensitivity following nerve injury. Furthermore, the dimethyl fumarate-induced antinociceptive activity was retained in wildtype mice, but lost in both male and female Nfe2l2^−/−^ mice with SNI as well as when treated with the Nrf2 inhibitor trigonelline [144]. It is important to note that besides targeting Nrf2, dimethyl fumarate and its metabolite monomethyl fumarate are potent agonists for the hydroxycarboxylic acid receptor 2 (HCAR2) and are approved for the treatment of multiple sclerosis [156,157,158]. HCAR2 also regulated neuropathic pain plasticity in CCI and SNI models of neuropathic pain and dimethyl fumarate alleviated the neuropathic pain-induced hypersensitivities [159].

Li et al. reported a significant decrease in Nrf2 protein in the medial prefrontal cortex (mPFC), hippocampus, spinal cord, and skeletal muscle, but not in the nucleus accumbens in SNI rats with an anhedonia (loss of pleasure as measured by the sucrose preference test) phenotype compared to the sham or anhedonia-resistant rats. The expression of Keap1 was also decreased in mPFC, hippocampus, and muscle of anhedonic rats compared to the sham or anhedonia-resistant rats. Nrf2 inducer sulforaphane alleviated SNI-induced mechanical thresholds by normalizing Keap1-Nrf2 signaling in the spinal cords of anhedonia-susceptible animals but did alter signs of anhedonia. However, sulforaphane did improve mechanical thresholds and anhedonia when administered 30 min prior to SNI. The study provides solid evidence of the beneficial effects of sulforaphane in SNI animals via normalizing the decreased Keap1-Nrf2 signaling (Table 5) [145].

#### 2.3.5. Spinal Nerve Ligation (SNL)

Kim and Chung developed the classical spinal nerve libation model (SNL) model in which the unilateral L5 and/or L6 spinal nerves of rodents at a distal location from the dorsal root ganglion were tightly ligated [160]. This model simulates human causalgia [160]. In rodents with SNL, allodynia and hypersensitivity develop quickly after the ligation and persist at least 4 months [161]. Li et al. reported that mice with SNL exerted impaired autophagy, which led to an increase in neuroinflammation via the TRAF6-MAPK8-NF-κB pathway [162]. The impaired autophagy reduced the protective effects of astrocytes on oxidative stress in neurons by reducing the release of glutathione and, thus, maintaining neuropathic pain sensitivity. Reported also was the synergistic activation of autophagy and the NFE2L2 pathway, which induced stronger analgesic activity as compared to the activation of autophagy alone [162]. Ren et al. reported that dimethylitaconate, a derivative of itaconate, reduced neuroinflammation, decreased phosphorylation of ERK1/2, and increased the level of Nrf2 in the DRG and spinal cord of SNL mice (Table 5) [146].

## 3. Conclusions and Future Directions

The preclinical studies presented in this review highlight the therapeutic potential of Nrf2 inducers for alleviating neuropathic pain in several neuropathy conditions modeled in rodents. Collectively, the conclusion of these studies is that Nrf2 signaling activation leads to alleviation of ROS-activated oxidative stress, neuroinflammation, and mitochondrial dysfunction (see working model illustrated in Figure 1). However, there are a few aspects, including sex differences and limited clinical studies on Nrf2-induced alleviation of neuropathic pain, that require further investigations. Further, the protective effects of Nrf2 against specific cell types and its neuroanatomical location still need to be determined. We have singled out a few mechanisms underlying the activation of Nrf2 signaling pathways by different natural and/or synthetic Nrf2 inducers. Together, the data suggest that pharmacological activation of Nrf2 can ameliorate neuropathic pain conditions in preclinical studies, indicating that Nrf2 inducers can be used as an adjuvant therapy to alleviate neuropathic pain conditions. However, a better understanding of redox biology under chronic pain and the treatment of chronic pain by antioxidant therapy can be enhanced by further exploring the mechanisms of action of Nrf2 inducers in different neuropathic pain conditions.

Unfortunately, we found that most of the cited studies focused on the effects of Nrf2 signaling in male rodents. The importance of the sex differences underlying the chronic pain mechanisms is well established. In humans, females are more prone to encountering chronic pain as compared to males [163,164]. Furthermore, female patients are reported to have higher pain scores on the visual analog scale than male patients [165]. Despite these robust sex differences in response to pain treatments, the studies of both clinical and preclinical sex differences are underpowered [166]. Therefore, future studies should delve into the mechanisms of action of Nrf2 in female subjects both under in vivo and clinical settings and elucidate any sex differences in Nrf2-mediated amelioration of pain.

Collectively, the present review provides mechanistic insights into the involvement of Nrf2 signaling and its activation by different inducers in preclinical studies and lists the potential future directions that will help to design and optimize Nrf2 inducers that will activate the Nrf2 signaling pathways and to ameliorate neuropathic pain conditions. Nrf2 inducers are currently being examined in several clinical trials for inflammatory lung conditions (NCT01335971, NCT04937855, NCT0315665), osteoarthritis (NCT04638387), and cancer (NCT03182959, NCT03872427. NCT04265534). Data coming out of these clinical trials indicate that Nrf2 inducers possess good efficacy, safety, and tolerability, and some of the Nrf2 inducers and/or signaling pathway are used and/or studied in clinical trials [167,168,169]. One clinical trial is also screening for tumor mutations in KEAP1 or NRF2/NFE2L2 genes (NCT04698681). In the current review, we found that a randomized controlled study reported that L-carnosine exerted neuroprotective activity by significantly decreasing proinflammatory (NF-κB, TNF-α) and apoptotic (caspase-3) markers and increasing Nrf2 in colorectal cancer patients with oxaliplatin-induced PN [107]. Therefore, the use of Nrf2 inducers in treating neuropathic pain under clinical settings holds great promise in the future.

## Figures and Tables

**Table 1:** Chemical structures of Nrf2 inducers.

5-fluoro-2-oxindole	Alphalipoic Acid	Bardoxolone Methyl	Berberine
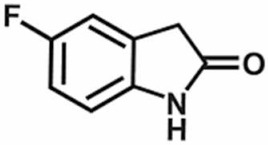	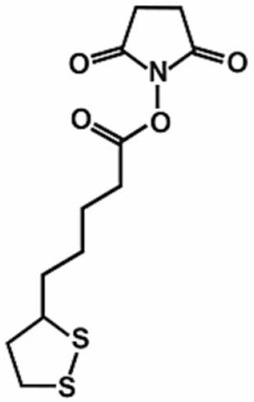	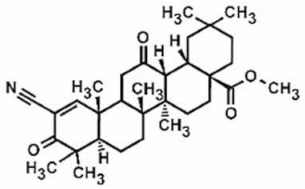	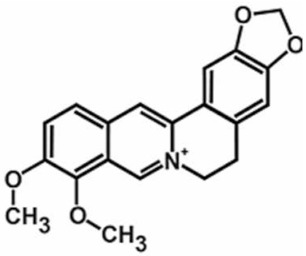
**Bergenin**	**Bromelain**	**Carvacrol**	**Curcumin**
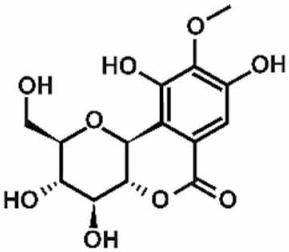	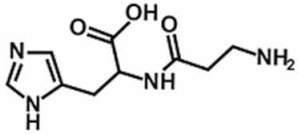	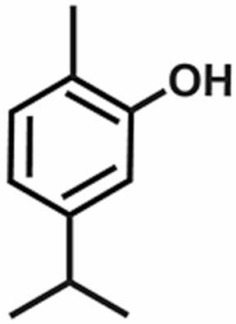	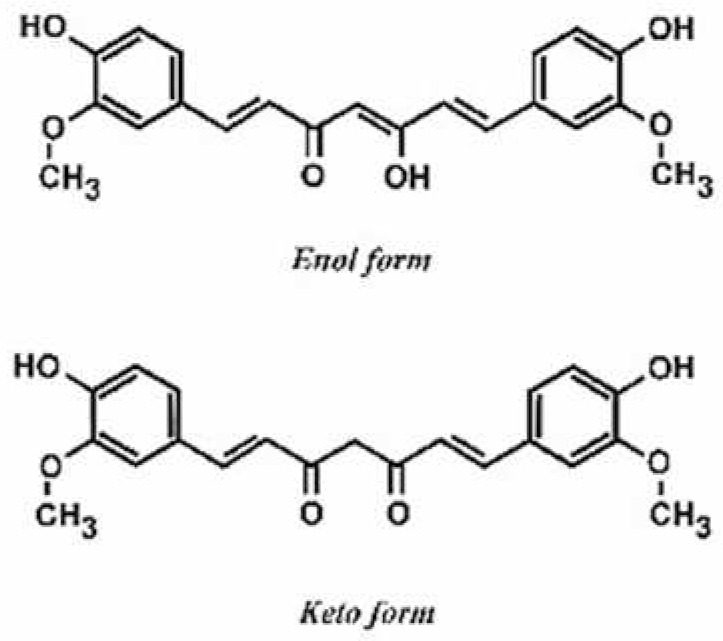
**Deguelin**	**Dexmedetomidine**	**Diallyl Disulfide**	**Diallyl Trisulfide**
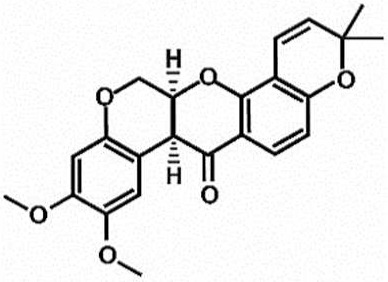	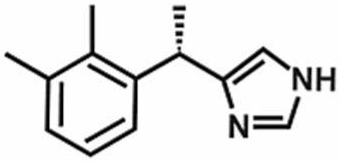	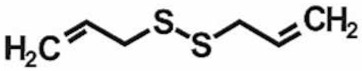	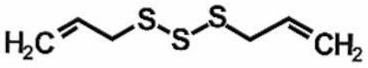
**Dimethyl Fumarate**	**Dimethylitaconate**	**Diosgenin**	**Diphenyl Diselenide**
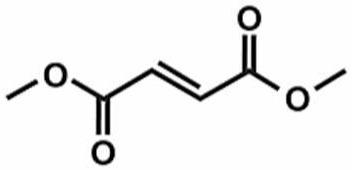	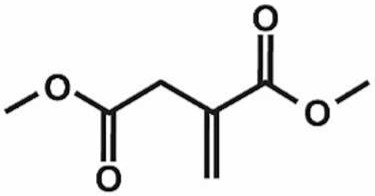	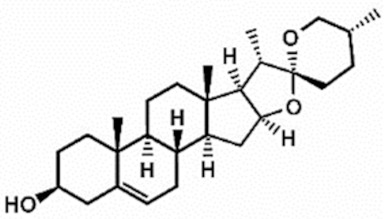	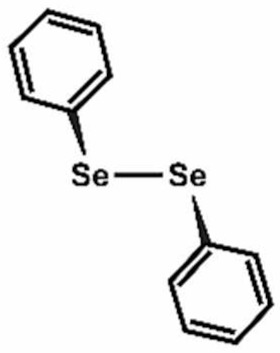
**Fisetin**	**Formononetin**	**Isoliquiritigenin**	**Isoquercitrin**
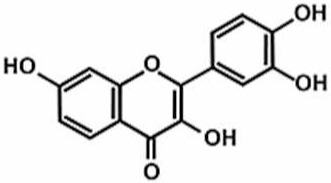	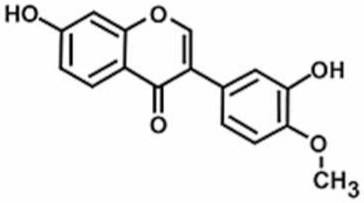	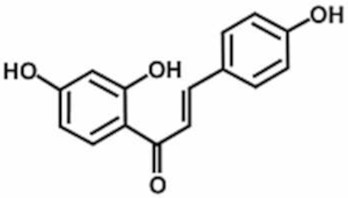	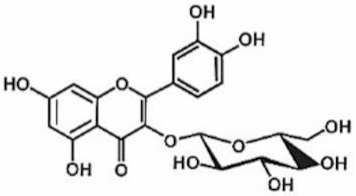
**L-carnosine**	**Levo-corydalmine**	**Mitoquinone**	**Monomethyl Fumarate**
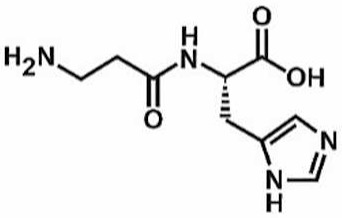	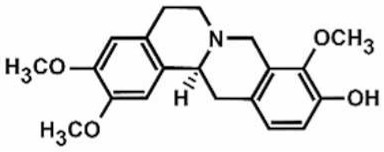	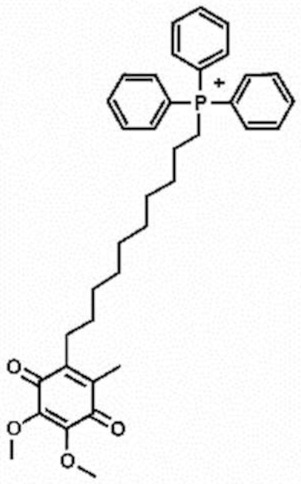	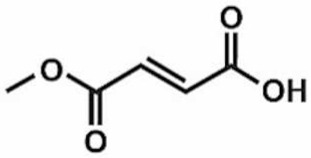
**Oleuropein**	**Oltipraz**	**Paeoniflorin**	**Plumbagin**
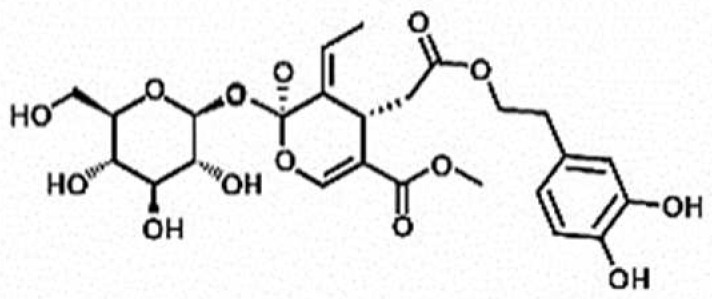	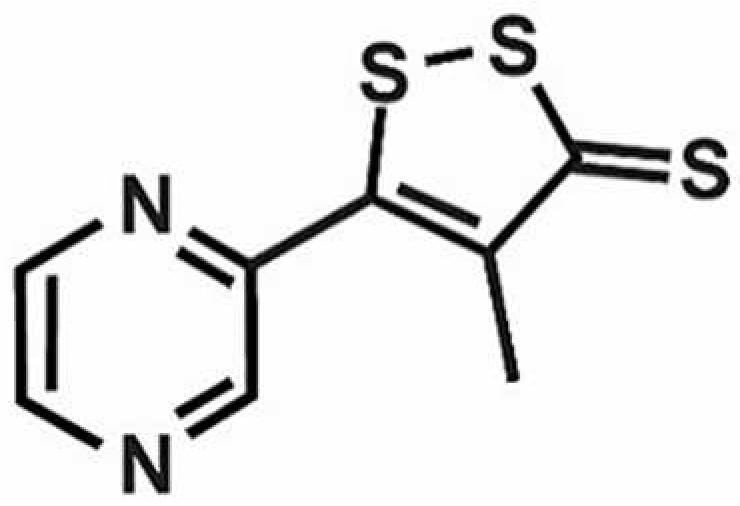	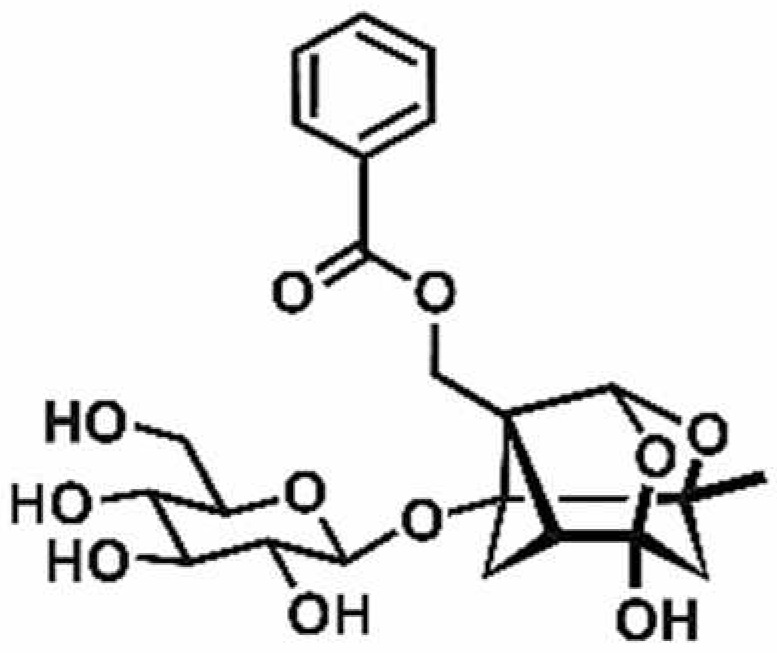	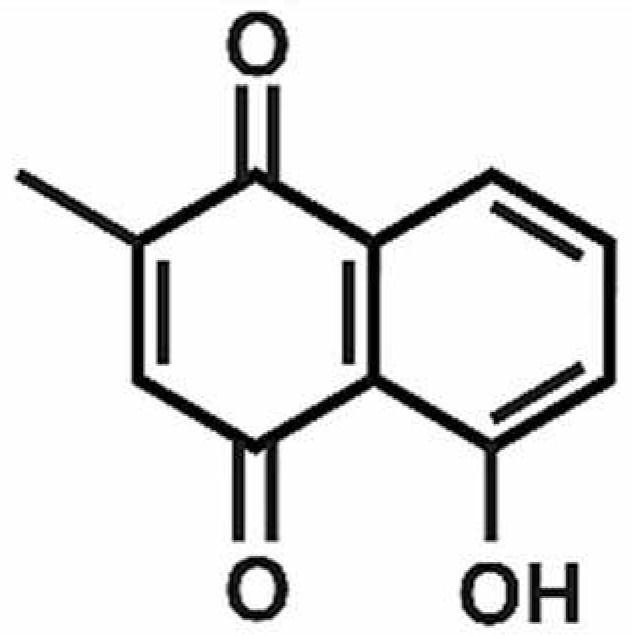
**Polydatin**	**Quercetin**	**Resveratrol**	**Rosiglitazone**
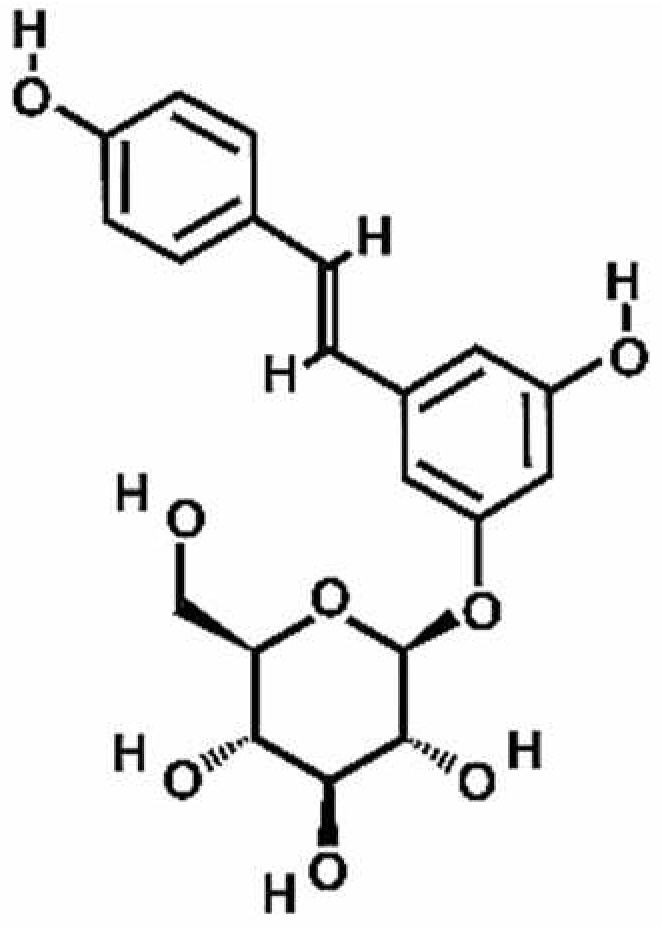	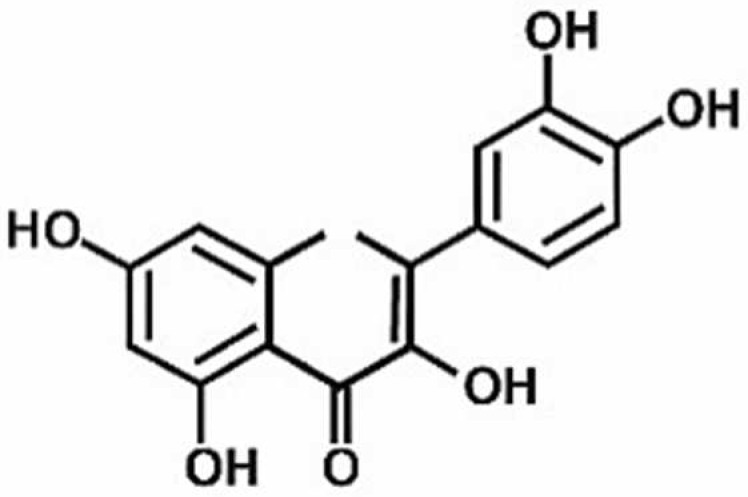	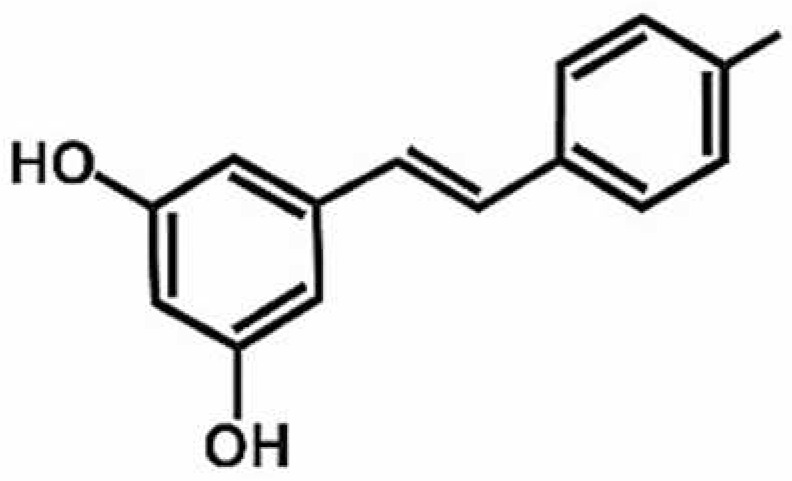	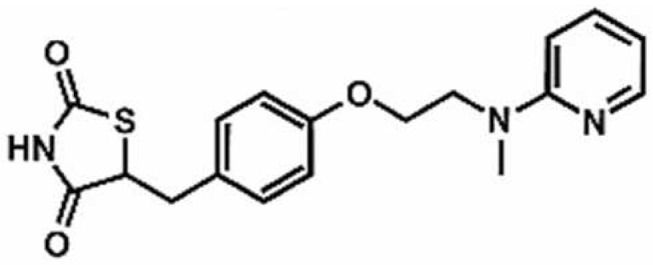
**RTA-408**	**Rutin**	**Sulforaphane**	**Tanshinone IIA**
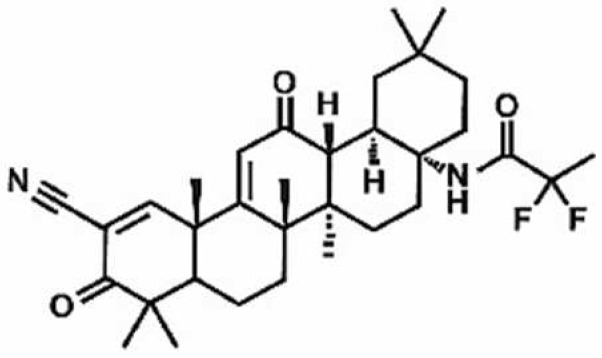	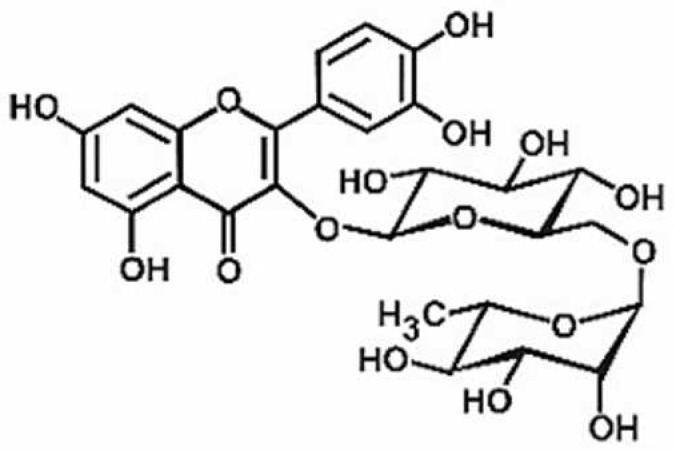	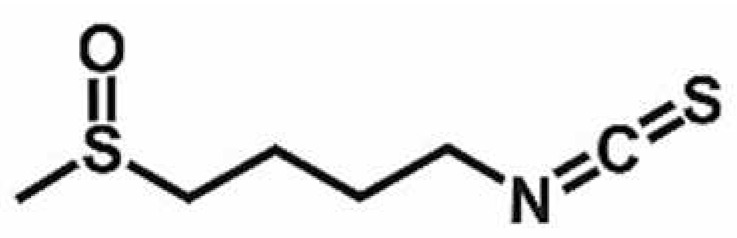	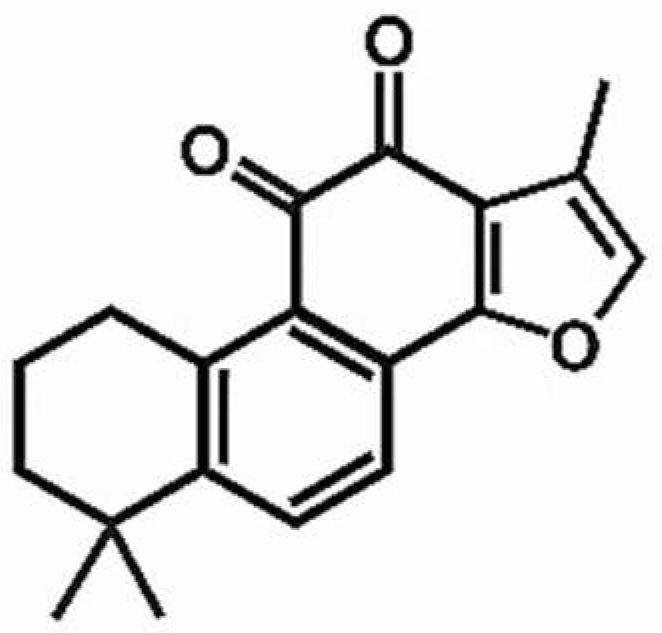
**Taurine**	**tBHQ**	**UFP-512**	
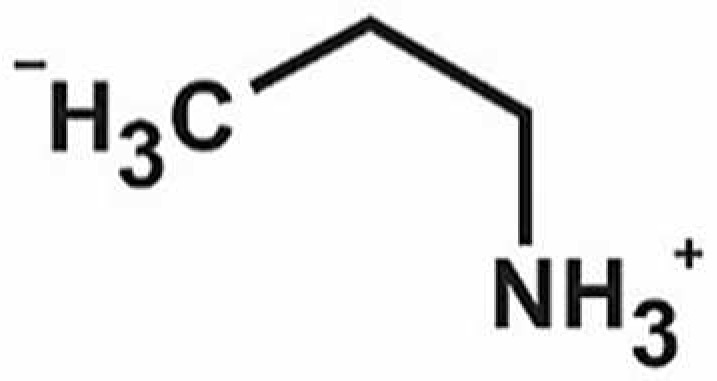	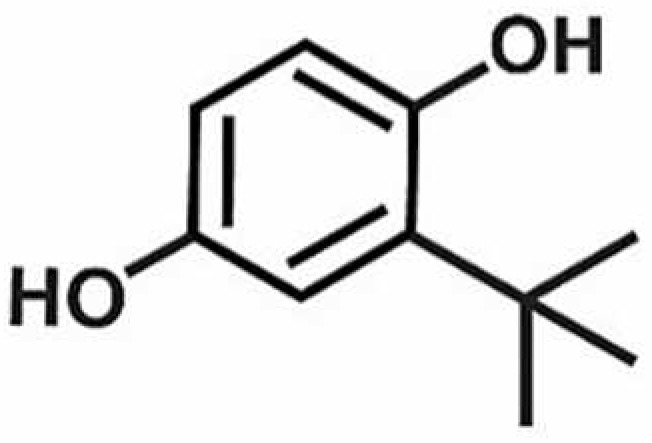	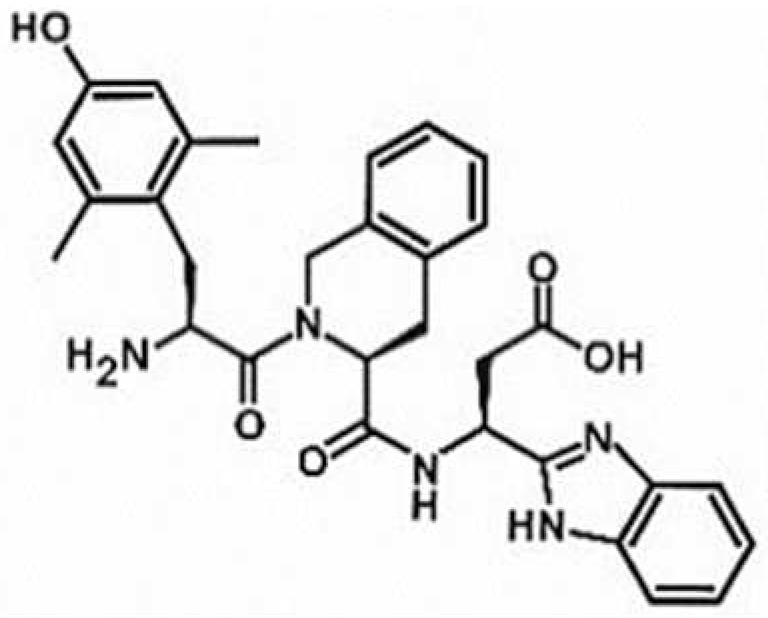	

Note. The chemical structures are listed in alphabetical order.

## Abbreviations

4-ANI	4-amino 1, 8-naphthalimide
8-iso	PGF2α 8-isoprostaglandin F2α
8-OHdG	8-hydroxy-2′-deoxyguanosine
AREs	antioxidant response elements
BDNF	brain-derived neurotrophin factor
BRD4	bromodomain-containing protein 4
BTB	bric-a-brac, tramtrack, broad-complex
bZip	basic region leucine zipper
CCI	chronic constriction injury
CIPN	chemotherapy-induced peripheral neuropathy
CoPP	cobalt protoporphyrin IX
COX-2	cyclooxygenase-2
Cul	cullin
DN	diabetic neuropathy
DOR	delta opioid receptor
DPDPE	[dPen(2),d-Pen(5)]-Enkephalin]
DRG	dorsal root ganglion
ECN	7β-(3-Ethyl-*cis*-crotonoyloxy)-1α-(2-methylbutyryloxy)-3,14-dehydro-*Z*-notonipetranone
ERK	extracellular signal-regulated kinase
GSH	glutathione
GSTP1	glutathione S-transferase pi 1
H_2_S	hydrogen sulfide
HCAR2	hydroxycarboxylic acid receptor 2
HO-1	heme oxygenase-1
i.p.	intraperitoneal
IL-1β	interleukin-1 beta
IL-6	interleukin-6
JNK	c-Jun N-terminal kinase
Keap1	Kelch-like ECH-associated protein 1
MAF	muscle aponeurosis fibromatosis
MCQ	mitochondrial quality control
mPFC	medial prefrontal cortex
NAD	nicotinamide adenosine dinucleotide
NF-κB	nuclear factor kappa B
NLRP3	NOD-like receptor protein 3
NOX4	nicotinamide adenine dinucleotide phosphate, reduced (NADPH) oxidase 4
NQO1	NAD(P)H:quinone oxidoreductase1
Nrf2	nuclear factor erythroid 2 (NFE2)-related factor 2 (Nrf2)
PARP	poly (ADP-ribose) polymerase
PEA	palmitoylethanolamide
PEA-OXA	2-pentadecyl-2-oxazoline of palmitoylethanolamide
PERK	protein kinase RNA-like endoplasmic reticulum kinase
PGC-1α	peroxisome proliferator activated receptor-gamma coactivator-1α
PN	peripheral neuropathy
PPARγ	peroxisome proliferator-activated receptor gamma
PSNL	partial sciatic nerve ligation
RNS	reactive nitrogen species
ROS	reactive oxygen species
s.c.	subcutaneous
SC	Schwann cell
SIRT	sirtuin
SNC	sciatic nerve crush
SNC-80	(+)-4-[(α(R)-α-[(2S,5R)-4-allyl-2,5-dimethyl-1-piperazinyl]-(3-methoxybenzyl)-N,N-diethylbenzamide]
SNI	spared nerve injury
SNL	spinal nerve ligation
SOD	superoxide dismutase
STZ	streptozotocin
TNF-α	tumor necrosis factor alpha
TRP	transient receptor potential
WT	wildtype
